# First person – Bilal Akhtar

**DOI:** 10.1242/bio.059225

**Published:** 2022-02-04

## Abstract

First Person is a series of interviews with the first authors of a selection of papers published in Biology Open, helping early-career researchers promote themselves alongside their papers. Bilal Akhtar is first author on ‘ A human stem cell resource to decipher the biochemical and cellular basis of neurodevelopmental defects in Lowe syndrome’, published in BiO. Bilal is a research fellow in the lab of Prof. Raghu Padinjat at the National Center for Biological Sciences, Bengaluru, India, investigating molecular mechanisms of neurodeveleopmental disorders using a stem cell based approach.


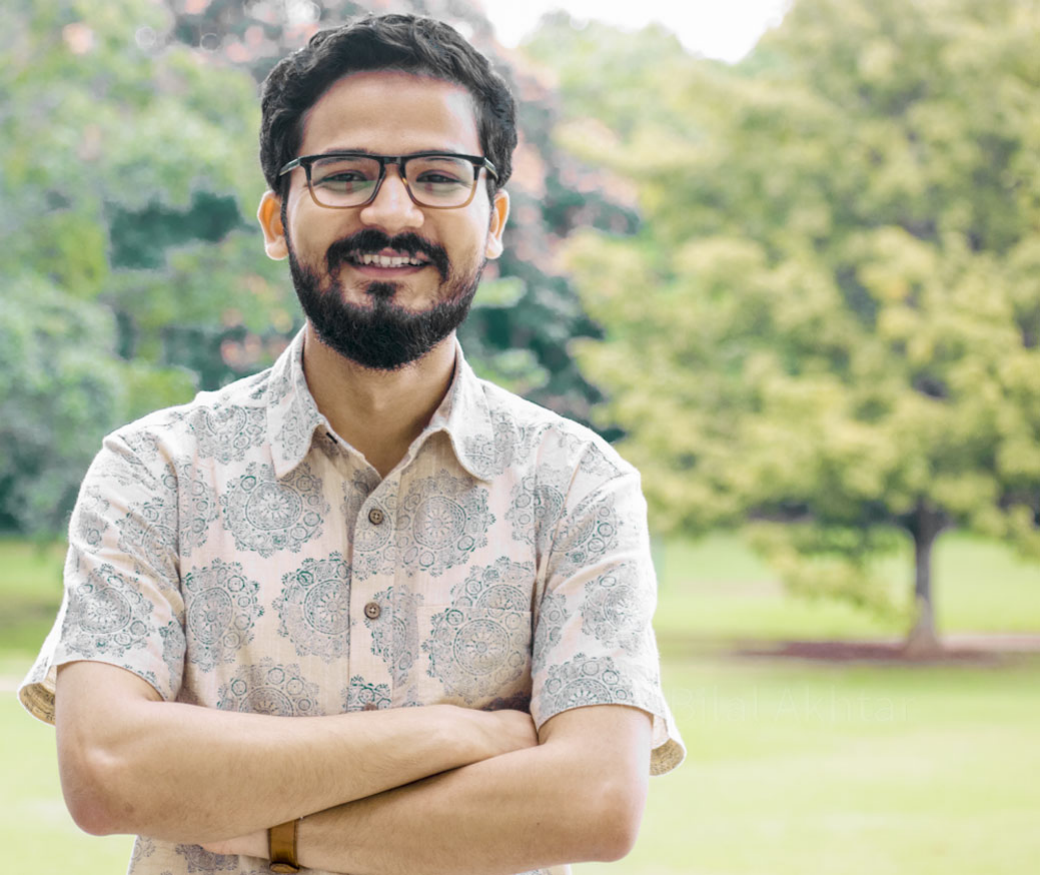


**Bilal Akhtar**

**What is your scientific background and the general focus of your lab?**

I obtained my dual master's in Bioinformatics and Applied Biotechnology from the Institute of Bioinformatics and Applied Biotechnology (IBAB) Bengaluru, India. Here I was introduced to CRISPR-Cas 9 genome engineering technique and use of bioinformatics to perform precision driven research. Following my interest in genetic engineering and disease modelling I joined Prof. Raghu Padinjat's lab at the National Center for Biological Sciences (NCBS), Bangalore as a research fellow. The lab focuses on function and signalling of phosphoinositides (PIs). My project involved generating a ‘disease-in-a-dish’ model for understanding the mechanism of Lowe syndrome using patient derived stem cells and neurons. Lowe syndrome is caused by loss of function of *OCRL* gene, which codes for a 5-phosphatase in the PI cycle.

**How would you explain the main findings of your paper to non-scientific family and friends?**

Our study can be broadly classified into two phases. Firstly, it involved generating the stem cell (iPSC and NSC) and neuronal model from patient samples, which in principle should mimic Lowe syndrome. Secondly, it involved investigating the biochemical and gene expression changes that occur in these models when compared to a healthy individual.

**What are the potential implications of these results for your field of research?**

Not only does our study circumvent the particular challenge in obtaining conventional biopsy samples from the human brain to study neurodevelopmental defects in Lowe syndrome patients by providing functional stem cell derived neuronal model but it also sheds light on biochemical changes that occur in the levels of phosphoinositides. Apart from this, our study provides valuable insight into the transcriptional changes of other 5-phosphatases in the background of loss of OCRL and how it affects the overall transcriptomic topology (RNA-Seq) of the cell.

**Figure:**
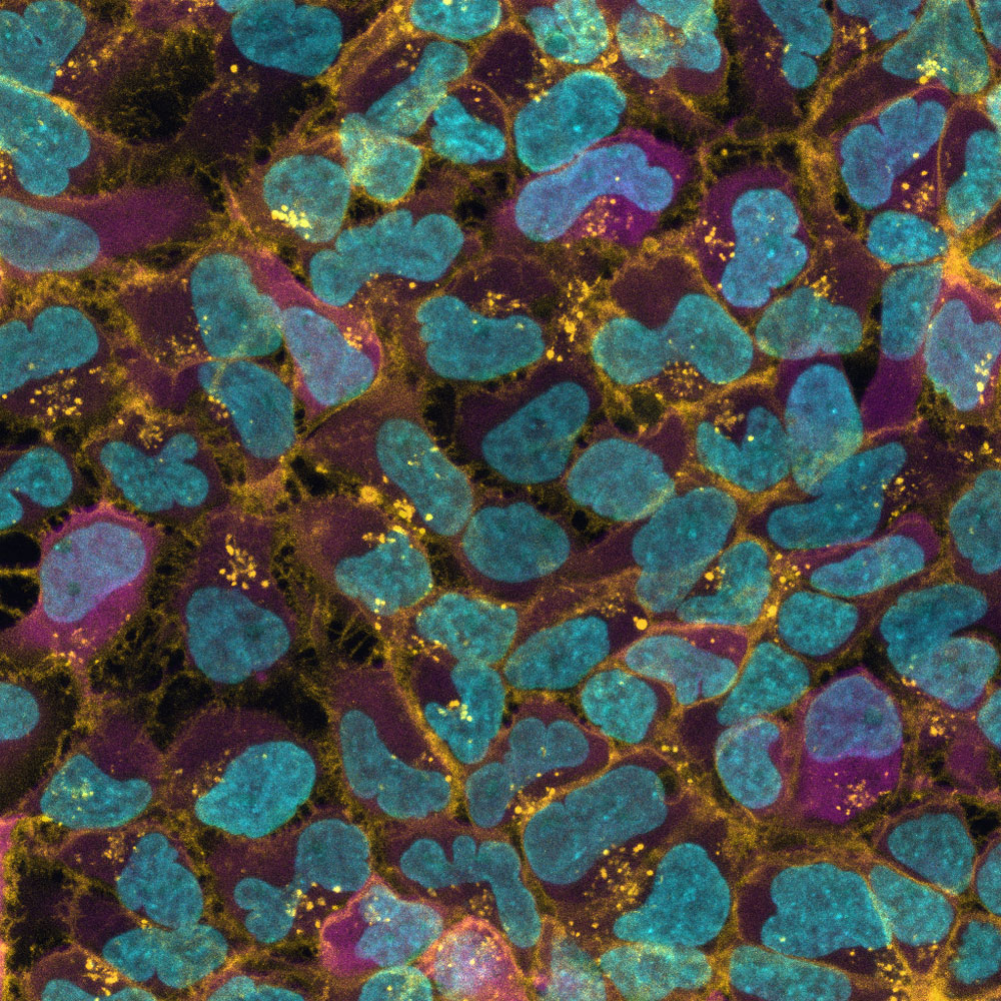
Genetically engineered Lowe syndrome patient neural stem cells (magenta) expressing PI(4,5)P_2_ biosensor (yellow) localized at the plasma membrane.

**What has surprised you the most while conducting your research?**

A multifaceted approach should be taken to answer any research question; the resolution at which one decides to investigate for the answer decides the outcome of their research. For example, as per our hypothesis the level of phosphatidylinositol 4,5-bisphosphate [PI(4,5)P2] should have been elevated in neural cells derived from Lowe syndrome patient, but the mass spectrometry data suggested otherwise. Later on when we investigated plasma membrane using biosensor for PI(4,5)P_2_ we did find relatively small but functional pool of PI(4,5)P_2_ elevated.

**What, in your opinion, are some of the greatest achievements in your field and how has this influenced your research?**

Our research stands on the shoulders of two giants that have immensely shaped the biological science research of the 21st century, the discovery of pluripotent factors by Dr Yamanaka's group and the advent of genetic engineering using CRISPR and TALENs. Direct implementation of these seminal discoveries in our project has led us a step closer to understanding the function of phosphoinositides in neuronal cell biology and brain disorders using human genetics and iPSC derived neural cell models.

**What changes do you think could improve the professional lives of early-career scientists?**

Personally speaking, I feel there is a discord between the way we are taught how science works in the classroom versus what we encounter when one starts working in a research lab. Hence, introduction to actual research should be part of the curriculum since day one and not merely for few months in a semester as part of the thesis. Apart from this, funding opportunities for early-career researchers and ease of accessibility to scientific papers (Open Access journals like Biology Open) especially for researchers in developing countries can go a long way in uplifting professional lives of early-career scientists.

**What's next for you?**

Upon completion of my project and submission of the manuscript, I am currently looking for doctoral positions in stem cell based translational research in the US and Europe.
